# A review of nonrevenue water assessment software tools

**DOI:** 10.1002/wat2.1413

**Published:** 2020-02-18

**Authors:** Taha M. AL‐Washali, Mohamed E. Elkhider, Saroj K. Sharma, Maria D. Kennedy

**Affiliations:** ^1^ Department of Civil Engineering and Geosciences Delft University of Technology Delft The Netherlands; ^2^ Environmental Engineering and Water Technology Department IHE Delft Institute for Water Education Delft The Netherlands; ^3^ Water and Environment Center Sana'a University Sana'a Yemen

**Keywords:** apparent losses, leakage, nonrevenue water, performance indicators, software tool

## Abstract

Several software tools are available that can assess the performance of nonrevenue water (NRW) in water distribution networks and plan for reduction measures. Of the 21 tools that have been reported in the literature, 12 are freely available. The creation of these many tools and different versions of each individual tool indicates the promising future of NRW software development. This review comprises 12 freely available tools for water balance establishment, NRW performance assessment, and NRW reduction planning. Most of the tools have been developed to establish standard annual water balances and recommended performance indicators (PIs) for the entire network. Some tools have been developed to intervene and reduce the leakage in a district metered area. Key features increasingly being included in NRW software include uncertainty analysis, recognition of supply intermittency, and accommodation of a guidance matrix and benchmarks. Leakage assessment is fully recognized, and leakage reduction analyses are increasingly growing in the software tools. However, much less attention has been paid to assessing and options for reducing apparent losses. Although a comprehensive NRW management tool for monitoring, planning, and intervention is not currently available, developing a comprehensive tool is worthwhile, in the form of one package or a kit of smaller tools. Toward this goal, the article provides insights and recommendations addressing topics of intermittency, normalization, multi‐method assessment, planning for the reduction of apparent and real losses, and estimation of the economic level of water loss.

This article is categorized under:

Engineering Water > Planning Water
Engineering Water > Methods

## Graphical abstract

Flowchart on the fit‐for‐context software tools for establishing water balance and performance indicators in water distribution networks.

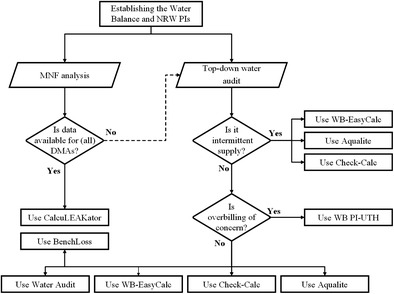

**Abbreviations**
AL: apparent losses
ALC: active leakage control (leak detection surveys)
AM: assets management
AWWA: American Water Works Association
CAL: component analysis of the leakage
CMIs: customer meter inaccuracies
DHEs: data handling errors
DMA: district metered area
ELL: economic level of leakage
FAVAD: fixed and variable area discharge
GWR: global water resources
MNF: minimum night flow
N_1_: pressure‐leakage relationship
N_2_: pressure bursts relationship
N_3_: pressure consumption relationship
NRW: nonrevenue water
P: pressure
PIs: performance indicators
PM: pressure management
PRVs: pressure reducing valves
RL: real losses
RTM: repair‐response time minimization
SIV: system input volume
T: supply time
UC: unauthorized consumption
WB: water balance
WRC: Water Research Commission
WRF: Water Research Foundation

## 1 INTRODUCTION

The collapse of water supply services negatively affects public health, the economy, education, women, human dignity, and triggers disease outbreaks. Water supply is crucial for a healthy and prosperous life. However, sustaining water services is challenging for every water utility. Water loss remains a major efficiency concern in all distribution networks, as it causes water wastage, technical burdens, water contamination, and revenue losses. The difference between the amount of water put into a distribution system and the amount of water billed to consumers is called nonrevenue water (NRW) (American Water Works Association, 2016; Lambert & Hirner, 2000). It has three components: real losses (RL), that is, leakages out of the distribution network; apparent losses (AL), which are the amount of water used by customers but not paid for—that is, unauthorized uses, customer meter inaccuracies, and data handling and billing errors; and unbilled authorized consumption (UAC), which includes other authorized uses by or via the utility but are not billed, such as water used for washing network pipes or for the fire department (AL‐Washali, Sharma, & Kennedy, 2018). The global volume of NRW is estimated to be 126 billion cubic meters annually, costing US $39 billion every year (Liemberger & Wyatt, 2018).

Nonrevenue water assessment basically involves quantifying losses in a particular system without considering where the losses are actually taking place (Puust, Kapelan, Savic, & Koppel, 2010). Firstly, the volume of NRW should be estimated. For an intermittent supply, NRW should be normalized. Normalization is a straightforward task when it involves adjusting the volume of NRW as the supply in the system is continuous (24/7; AL‐Washali, Sharma, Kennedy, AL‐Nozaily, & Mansour, 2019). Secondly, NRW should be broken down into components using different methods (AL‐Washali, Sharma, & Kennedy, 2016). Following that, NRW components and subcomponents can be prioritized for intervention measures and to minimize losses in the system (Al‐Washali et al., 2020; AL‐Washali, Sharma, Kennedy, et al., 2019). Partitioning the network into district metered areas (DMAs) yields enormous benefits including pressure control, network management, leakage monitoring, leakage detection, maintaining water quality, and asset management. There are several methods for network partitioning, based on criteria including topology, reachability, connectivity, redundancy, and network vulnerability (Deuerlein, 2008; Di Nardo, Di Natale, Santonastaso, & Venticinque, 2013; Galdiero, De Paola, Fontana, Giugni, & Savic, 2015; Morrison, Tooms, & Rogers, 2007). Network graph methods are common but other methods are also proposed (Deuerlein, 2008; Di Nardo, Di Natale, & Di Mauro, 2013; Galdiero et al., 2015; Kesavan & Chandrashekar, 1972; Perelman & Ostfeld, 2011). Incorporating the establishment of DMAs with pressure management is fruitful (Alonso et al., 2000; Creaco & Pezzinga, 2014; De Paola et al., 2014). Pressure management is an effective measure to reduce leakage. It depends on the proper usage and location of pressure reducing valves (PRVs; Alonso et al., 2000; Araujo, Ramos, & Coelho, 2006; Creaco & Pezzinga, 2014; Dai & Li, 2014; Page, Abu‐Mahfouz, & Mothetha, 2017; Vicente, Garrote, Sánchez, & Santillán, 2016). Active leakage detection and control uses methods to detect, locate, and pinpoint leaks (Li, Huang, Xin, & Tao, 2014; Puust et al., 2010; Wu & Liu, 2017). Designing the frequency of leakage detection surveys based on economics has been suggested by Lambert and Fantozzi (2005); it is based on estimating the rate of rise of leakage (Lambert & Lalonde, 2005). However, leakage cannot be totally eliminated. The economic level of leakage (ELL) can be reached where the cost to further reduce leakage exceeds the expected benefits (Ashton & Hope, 2001; Kanakoudis, Tsitsifli, & Papadopoulou, 2012; Pearson & Trow, 2005). A similar concept applies for the economic level of AL (Arregui, Cobacho, Soriano, & Jimenez‐Redal, 2018). The result of combining the economic levels of AL and RL is the economic level of water loss. Besides the many software tools that simulate and hydraulically model the network pipes and appurtenances such as EPANET, WaterGYMS, InfoWater, WDNetXL, H_2_O MAPWater, and KYPIPE, there are many (commercial) tools particularly designed to assist water utilities assess their losses and plan reduction interventions (Halfawy & Hunaidi, 2008; Hamilton & McKenzie, 2014; Klingel & Knobloch, 2015; Liemberger & McKenzie, 2003; Sturm, Gasner, Wilson, Preston, & Dickinson, 2014; Tabesh, Yekta, & Burrows, 2009; Tsitsifli & Kanakoudis, 2010). This article, however, reviews 12 freely available software tools for water loss assessment, investigating their functionalities and limitations, and suggesting guidelines for their use and improvement. This will help software users familiarize themselves with these tools and their underlying concepts, and select the appropriate fit‐for‐purpose tool for each context. The future prospects for the industry are eventually highlighted.

## 2 NRW ASSESSMENT SOFTWARE TOOLS

While this article focuses on freely available NRW tools, some tools reported in the literature are commercially available. These include Aquadas‐QS (Aquadas‐QS, 2007), Aqualibre (Liemberger & McKenzie, 2003), Auditsolve (Sturm et al., 2014), SigmaLite (ITA, 2000), Leaks suite (Lambert, 2015b), Prototype (Halfawy & Hunaidi, 2008), NAIS (Heydenreich & Kreft, 2004), Netbase (Netbase, 2019), and ÖVGW spreadsheet (ÖVGW, 2009). The 12 freely available tools have been designed for water loss assessment, water balance establishment, and NRW PIs evaluation. Table 1 presents these tools and their approaches, which were developed in response to the establishment of standard terminology, standard water balance (WB) methods (Lambert & Hirner, 2000), and recommended NRW PIs (Alegre, Hirner, & Baptista, 2000). The main functionalities of the tools are: (a) the use of a top‐down water audit to estimate or assume AL, from which RL and NRW PIs are calculated. The top‐down water balance is usually conducted for a period of 1 year and encompasses the whole system (i.e., global); (b) the assessment of the RL based on the bottom‐up approach using minimum night flow (MNF) analysis in a DMA; and (c) the use of the burst and background estimates (BABE) for the whole network and in a DMA‐scale. Only one tool‐component analysis‐analyses different leakage reduction options. Table 1 summarizes these tools and their finicalities. Brief descriptions of each of these tools are also provided in a Supporting Information of this article.

**Table 1:** Free software tools for nonrevenue water assessment

#	Tool (version)	Reference	Developer	Environment	Description	Approach	Scale
1	AquaLite (v4.5)	(Mckenzie, 2007)	WRC	Windows‐based	A tool to establish WB and PIs	Top‐down	Global
2	AWWA Water Audit (v5)	(Water Loss Control Committee, 2014)	AWWA	Excel‐based	A tool to establish WB and PIs. Uses validity score (qualitative), not uncertainties	Top‐down	Global
3	BenchLeak	(Mckenzie, Lambert, Kock, & Mtshweni, 2002)	WRC	Excel‐based	A tool to establish WB and PIs	Top‐down	Global
4	BenchLoss (v2a)	(GWR‐Ltd, 2008)	GWR	Excel‐based	A tool to establish WB and PIs	Top‐down	Global
5	CalacuLEAKator (v4.3)	(Koldžo & Vucˇijak, 2013)	Djevad Koldzo	Excel‐based	A tool to establish WB and PIs, based on MNF analyses	MNF	DMA, global
6	CheckCalcs (v6b)	(Lambert, 2015a)	ILMSS Ltd.	Excel‐based	A tool to establish WB and PIs. Provides insights on leakage relationships, N_1_, N_2_, N_3_	Top‐down	Global
7	Component Analysis	(Sturm et al., 2014)	WRF	Excel‐based	WB and PIs are inputs. Analyses potential of leakage reduction interventions	Top‐do., BABE, PM, ALC	Global
8	EconoLeak (v1a)	(Mckenzie & Lambert, 2002)	WRC	Excel‐based	A tool to establish the ELL with cost–benefit analysis of ALC	ELL	Global
9	PresMac (v4.4)	(Mckenzie & Langenhoven, 2001)	WRC	Windows‐based	Operational tool for pressure management in a DMA, using PRVs	BABE, FAVAD, PRVs	DMA
10	SanFlow (v4.6)	(Mckenzie, 1999)	WRC	Windows‐based	A tool to model MNF in a DMA and breakdown leakage into components	MNF, BABE	DMA
11	WB‐EasyCalc (v5.16)	(Liemberger and Partners, 2018)	Roland Leimberger	Excel‐based	A tool to establish WB and PIs. Analyses impacts of changes in pressure, SIV, and supply time	Top‐down	Global
12	WB‐PI Calc‐UTH (v2.2)	(Tsitsifli & Kanakoudis, 2010)	Tsitsifli and Kanakoudis	Excel‐based	A tool to establish WB and PIs. Considers the overbilling practices in the balance	Top‐down	Global

Abbreviations: ALC, active leakage control; AWWA, American Water Works Association; BABE, burst and background estimates; DMA, district metered area; ELL, economic level of leakage; FAVAD, fixed and variable area discharge; GWR, global water resources; MNF, minimum night flow; PRV, pressure reducing valve; SIV, system input volume; WRC, Water Research Commission; WRF, Water Research Foundation.

## 3 NRW ASSESSMENT

**Figure 1:**
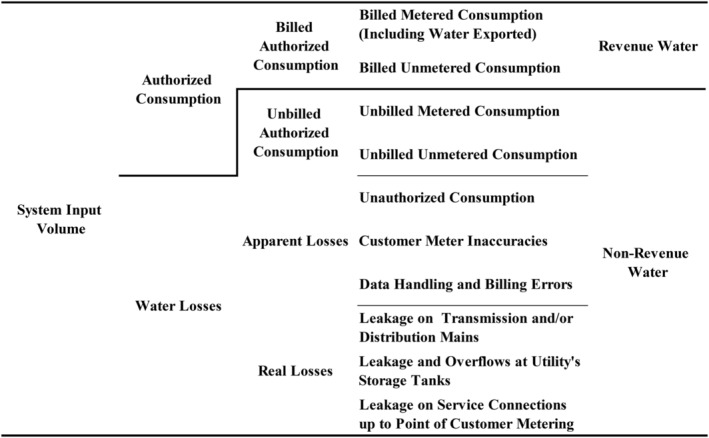
IWA Standard water balance (Lambert & Hirner, 2000). IWA, International Water Association

Lambert and Hirner (2000) suggested the standard terminology for a standard water balance in water distribution networks (Figure 1). Deducting the volume of billed consumption (BC) from the system input volume (SIV) gives the volume of the NRW. Deducting the volume of the UAC from NRW gives the volume of water loss. Breaking down water loss into AL and RL involves four methods. The top‐down methods start by estimating the volume of AL and then calculating the volume of RL. The bottom‐up methods analyze the leakage volume based on field measurements or available records. In the top‐down water audit, the AL components are estimated. To determine customer meter inaccuracies, a representative sample is tested in the laboratory at different flow rates that represent the field conditions (Arregui, Cabrera, Cobacho, & García‐Serra, 2006; Arregui, Cabrera Jr, & Cobacho, 2007; Walter, Mastaller, & Klingel, 2018). Data handling and billing errors are estimated by investigating historical billing records and trends (AWWA, 2016; Mutikanga, Sharma, & Vairavamoorthy, 2011). Estimating the amount of unauthorized use is challenging, and therefore it is commonly assumed arbitrarily (Al‐Washali et al., 2020; AWWA, 2016; Klingel & Knobloch, 2015; Mutikanga et al., 2011; Seago, Bhagwan, & McKenzie, 2004; Vermersch et al., 2016). After estimating the components of AL, RL can be calculated. Afterward, the International Water Association (IWA) standard water balance in Figure 1 can be established. Another top‐down method is the water and wastewater balance method (AL‐Washali et al., 2018), which assumes that AL enters the sewer network. Analyzing the WWTP inflows and comparing it to the BC enables the estimation of the volume of AL, from which RL are then calculated (AL‐Washali et al., 2018; Al‐Washali et al., 2020). These calculations establish the IWA water balance, after which best practice NRW PIs can be calculated for target monitoring and leakage benchmarking (Alegre et al., 2000; Alegre et al., 2016). Table 2 shows the recommended key PIs of NRW. Historically, the fundamental indicator for monitoring and benchmarking NRW was presenting NRW as a percentage of the SIV, using Equation (1). Nevertheless, consistent feedback from field data revealed that relying on this indicator for monitoring and benchmarking NRW progress is rather misleading. This is because it is strongly influenced by water consumption (Lambert et al., 2014), favors less water supply over more supplied water (AL‐Washali, Sharma, AL‐Nozaily, Haidera, & Kennedy, 2019), a zero‐sum indicator for BC, NRW, and SIV (Lambert, 2019), and because when used, the denominator in the first part of Equation (1) should be a cause of change in the numerator (Alegre et al., 2016). Tackling this problem, the PIs in Table 2 were proposed, to give meaningful input and inform perception about the status of NRW progress. The units of the PIs in Table 2 indicate vividly the intuition of each indicator and how it should be calculated. For the infrastructure leakage index (ILI), it can be calculated based on Equations 2, 3 (Lambert et al., 2014).

(1) $$\mathrm{NRW}\;\left(\%\right)=\frac{\mathrm{SIV}\;\left(m^{3}/\text{year}\right)-\mathrm{BC}\;\left(m^{3}/\text{year}\right)}{\mathrm{SIV}\;\left(m^{3}/\text{year}\right)}=\mathrm{AL}\;\left(\%\right)+\mathrm{RL}\;\left(\%\right)+\mathrm{UAC}\;\left(\%\right)$$

(2) $$\mathrm{ILI}=\frac{\text{CARL}}{\text{UARL}}$$

(3) $$\text{UARL}\;\left(\frac{\text{Litres}}{\text{serv}.\text{conn}.\;\mathrm{day}\;}\right)=\left(18\times \frac{L_{m}}{N_{c}}+0.80+0.025\;L_{P}\right)\times P_{\mathrm{ave}}$$

where NRW (m^3^/year) is nonrevenue water, SIV (m^3^/year) is the system input volume, BC (m^3^/year) is the billed consumption, AL (%) is the apparent losses, RL (%) is the real losses, UAC (%) is the unbilled authorized consumption, ILI (unitless) is the infrastructure leakage index, CARL is the current annual RL (m^3^/year), UARL (m^3^/year) is the unavoidable annual RL, *N*_c_ is the number of service connections, *L*_m_ is the length of mains in km, *L*_p_ is the length of private underground connection pipe in m between the edge of the street and the customer meters, and *P*_ave_ is the network's average operating pressure.

**Table 2:** Nonrevenue water key performance indicators

Level	Function		Performance indicator		
			Service connection density		
			>20/km of mains	<20/km of mains	Comments
1—Basic	Financial	NRW	Volume of NRW as % of SIV	Volume of NRW as % of SIV	Simple, not recommended
1—Basic	Operational	AL	m^3^/serv. conn./year	m^3^/km of mains/year	For target setting, not comparing systems
1—basic	Operational	RL	L/serv. conn./day	L/km of mains/day	For target setting not comparing systems
1—Basic	Operational	RL	L/serv. conn./day w.s.p.	L/km of mains/day w.s.p.	Allows for intermittent supply situations
2—Interm.	Operational	RL	L/serv. conn./day/m pressure	L/km of mains/day/m pressure	Useful for comparing systems
3—Detailed	Financial	NRW	Value of NRW as % of annual cost	Value of NRW as % of annual cost	Allows different unit costs
3—Detailed	Operational	RL	ILI	ILI	Powerful for comparing systems

Source: Alegre et al. (2016).

Abbreviations: AL, apparent losses; ILI, infrastructure leakage index; NRW, nonrevenue water; RL, real losses.

On the other hand, the bottom‐up methods are only for estimating the RL. MNF analysis is carried out for a DMA during night time when most customers are inactive. Flow and pressure measurements are analyzed and night flows should indicate the volume of the RL (Farley & Trow, 2003; Puust et al., 2010). The leakage rate can be estimated through this method using Equations 4, 5, 6 (Alkasseh, Adlan, Abustan, Aziz, & Hanif, 2013; AL‐Washali, Sharma, Kennedy, et al., 2019; Farley & Trow, 2003). Notably, this method can only be carried out for a DMA and scaling it up for the entire network is very uncertain (Al‐Washali et al., 2020; AL‐Washali, Sharma, Kennedy, et al., 2019).

(4) $$L_{\mathrm{DMA}@t_{\mathrm{MNF}}}=Q_{\mathrm{MNF}}-Q_{\mathrm{LNC}}$$

(5) $$Q_{\mathrm{DMA};\text{daily}}=L_{\mathrm{DMA}@t_{\mathrm{MNF}}}\times F_{\mathrm{ND}}$$

(6) $$F_{\mathrm{ND}}=\sum_{i=1}^{24}{\left(P_{i}/P_{\mathrm{MNF}}\right)}^{N_{1}}$$

where *L*_DMA@t‐MNF_ is the leakage rate in the DMA (m^3^/hr) at the time hour of MNF, *Q*_MNF_ is the minimum flow rate, *Q*_LNC_ is the legitimate night consumption in the DMA at the MNF time, *Q*_DMA‐daily_ is the daily leakage in the DMA, *F*_ND_ is the night day factor, and *i* is hours of the day.

After the RL are estimated through one of the above methods, it can be broken down into its subcomponents, using the BABE analysis (Lambert, 1994). Although most RL are avoidable, some are unavoidable, even in a new and well‐constructed network. Background leaks may be too small to detect by the available detection technology. In contrast, bursts are big enough to be reported for repair by customers or by the utility crew. Unreported bursts are usually detected by the leakage detection surveys (i.e., the active leakage control—ALC; AWWA, 2016; Puust et al., 2010). Unavoidable annual RL (UARL) can be estimated using a recommended empirical equation presented in Equation (3) (Lambert et al., 2014; Lambert, Brown, Takizawa, & Weimer, 1999). The BABE analysis is useful because it enables water utilities to understand the nature of RL and plan reduction measures.

Finally, because the water balance is associated with uncertainties, it is usually accompanied by uncertainty analysis (Lambert et al., 2014; Thornton, Sturm, & Kunkel, 2008). The uncertainties of the water balance can be calculated straightforward using the error propagation theory (Taylor, 1997). As the water balance problem is a process of adding and subtracting, the general equation of the error propagation theory in Equation (7) can be simplified as in Equation (8). The error propagation analysis is simple and sufficient for the water balance problem. It produces the same results with other advanced methods (Al‐Washali et al., 2020) such as Monte Carlo simulation (Rubinstein & Kroese, 2016). However, some of the tools do use the variance analysis based on the statistical principles of the root‐mean‐square method for the normally distributed data (Thornton et al., 2008), which generates the same uncertainties. In this case, the higher the variance of the water balance component, the more significant its uncertainty becomes. The variance for each water balance component can be calculated using Equation (9) for Gaussian distribution whose curve density is represented by Equation (10) (Thornton et al., 2008).

(7) $$\Delta Z=\sqrt{{\left(\mathrm{\delta Z}/\mathrm{\delta X}\right)}^{2}\;{\left(\Delta X\right)}^{2}+{\left(\mathrm{\delta Z}/\mathrm{\delta Y}\right)}^{2}\;{\left(\Delta Y\right)}^{2}}$$

(8) $$\Delta Z=\sqrt{\;{\left(\Delta X\right)}^{2}+{\left(\Delta Y\right)}^{2}}$$

where *X* and *Y* are independent and measurable quantities that are used to obtain a value of a calculated quantity *Z*; δ*Z*/*δ* is the partial derivative of the variable *Z* with respect to an independent parameter (*X* or *Y*), and Δ*X* and Δ*Y* are the uncertainties of the variables *X* and *Y*.

(9) σ2=Qm3year×Z1.962

(10) $$F\left(x;\mu ,\sigma^{2}\right)=\frac{1}{\sigma \sqrt{2\pi }}e^{-\frac{1}{2}{\left(x-\mu /\sigma \right)}^{2}}$$

where *σ* ^2^ is the variance, *Q* is the amount of the water balance component (m^3^/year), *Z* is the 95% confidence limit, *μ* is the mean, and *σ* is the standard deviation.

## 4 TOOLS FOR WATER BALANCE ESTABLISHMENT

Tables 1 and 3 shows that nine tools are basically water balance tools: AquaLite, AWWA Water Audit, BenchLeak, BenchLoss, CalcuLEAKator, CheckCalcs, Component Analysis, EasyCalc, and WB‐PI Calc‐UTH. The main focus of these tools is to establish the standard water balance and NRW PIs. Basic system data such as number of service connections, mains, and pressure data are input as well as the water balance data, and the main output is the standard water balance and NRW PIs. However, some tools have more or deeper features. EasyCalc remains the most detailed (Table 4), straightforward and comprehensive tool for the water balance establishment. It contains detailed input for system data, pressure data, water balance data, a historical comparison of water balances, and brief what‐if scenarios. CheckCalcs, AquaLite, and BenchLoss come next in tolerating essential details about a particular case study. AWWA Water Audit and Component Analysis are tools that are more standardized for water utilities in USA and North American countries, where the input of key figures and water balance components are briefly condensed in a sole or limited input. The limited input has eventually an impact on the sensitivity and the accuracy of the tool. However, the tools (AWWA Water Audit and Component Analysis) have complementary mini‐tools for data report, collection and validation. Similarly, WB‐PI Calc‐UTH and BenchLeak are tools that are locally focused. BenchLeak is one of the first water balance tools and now, is generally outdated and substituted by AquaLite. While all the water balance tools use only the recommended IWA PIs for NRW, WB‐PI Calc‐UTH has 170 PIs that cover broad aspects of the water service in general. It also has a unique feature of recognizing the overbilling practice, which overestimates the BC and subsequently underestimates the NRW. This is the impact of charging a customer a minimum BC (e.g., 10 m^3^/month) even though a customer does not consume this amount. The impact of this overbilling practice occurs only if the billing system cannot charge the monetary minimum consumption bill unless the volumetric real data in the billing system is altered (manually or automatically; AWWA, 2016). This process is rather destructive for essential costly data of a water utility and causes avoidable opacity of the utility performance. However, when this practice exists in a water utility, the overbilling should be considered in the standard water balance itself and this is the intuition of the WB‐PI Calc‐UTH tool (Tsitsifli & Kanakoudis, 2010).

**Table 3:**
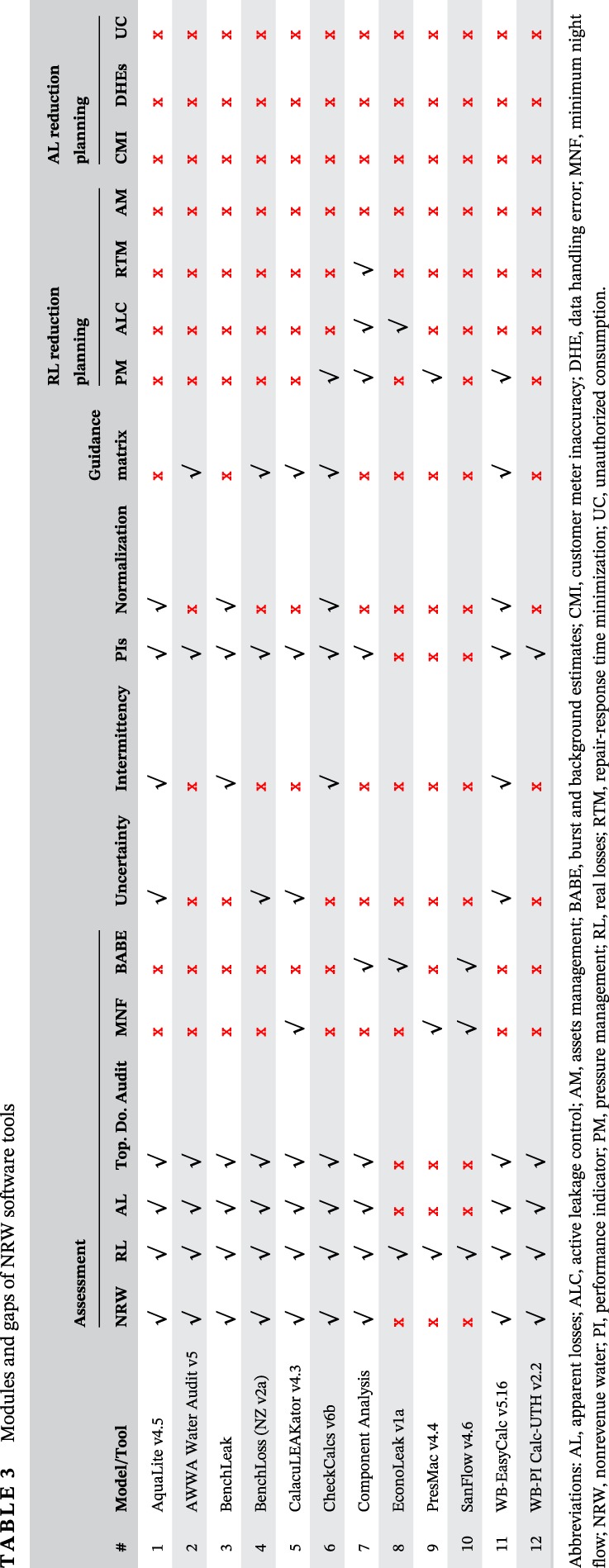


**Table 4:**
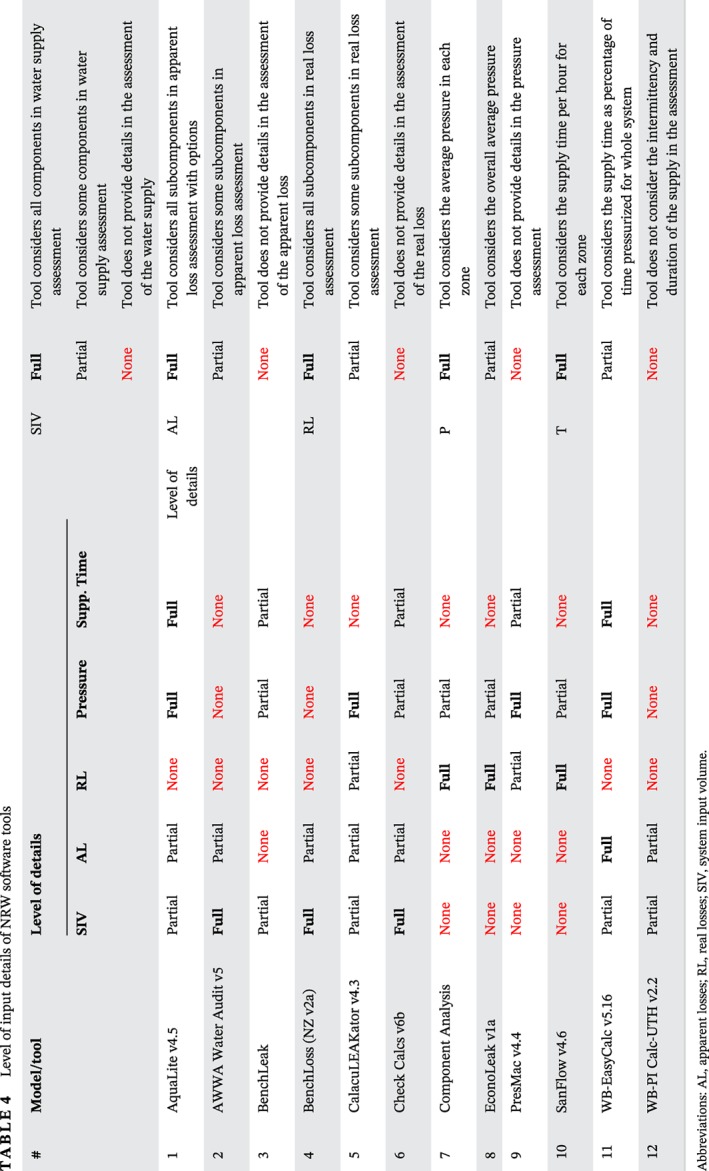


### 4.1 Intermittency and normalization

Another key issue is the applicability of these tools in intermittent water supply systems, where water is not available in the network 24/7 and customers adapt to this situation by setting up local storage tanks in their premises. In such a case, the volume of water loss is highly influenced by the volume of the supplied water. The greater is the volume of the water supplied into the network, the more will be the volume of water losses and the higher will be the PIs of NRW, indicating worse performance while it is not necessarily the case. To tackle this issue, the volume of water loss and its PIs have to be normalized and adjusted as if the supply is continuous (AL‐Washali, Sharma, AL‐Nozaily, et al., 2019). This normalization process enables monitoring and benchmarking the performance of water loss management in intermittent supply, which is an issue of increasing interest. However, only four tools recognize the intermittency in the tools' input: AquaLite, BenchLeak, CheckCalcs, and EasyCalc, where AquaLite and EasyCalc are relatively more detailed. Although normalizing the volume of NRW, AL and its PIs for monitoring and benchmarking is intuitive and critical, it is still a recent highlight that is not considered yet in the four tools. These tools normalize only the RL, explicitly in EasyCalc and AquaLite, and implicitly in CheckCalcs.

### 4.2 Uncertainty analysis

Introducing the uncertainties of the water balance components to the users of the tools is a significant achievement in raising the awareness of the water balance limitations and the practical way to improve them. The uncertainty analysis points out which input data should be more verified in order to minimize the uncertainty of the interesting output. AquaLite, BenchLoss, CalcuLEAKator, and EasyCalc use the variance analysis (Equation 9) for identifying the uncertainty of the water loss components. AquaLite has this feature for the water balance components but also for NRW PIs, which is an important gesture of AquaLite. CheckCalcs use the uncertainty analysis, with a similar approach to error propagation theory, to generate uncertainties for identifying the opportunity of pressure management and its influence on reducing the leaks, the bursts, and the consumption (N_1_, N_2_, and N_3_, respectively). It is, however, a key limitation for CheckCalcs and the remaining tools that they do not recognize the uncertainties of the water balance because the water balance is critically influencing all aspects of water loss management intervention and has a great implication on the estimated benefits of each intervention (AL‐Washali, Sharma, Kennedy, et al., 2019). It should be noted that CheckCalcs is just one free tool of many commercial tools that form one package (LeaksSuit) for leakage management.

Relevant to the uncertainty analysis is the use of validation score for the input and output of the tool, to determine the validity and reliability of the tool's output. This is a unique feature of the AWWA Water Audit whose validity score triggers changes in data acquisition rules when a low validity score is recorded. It is equivocal why AWWA Water Audit incorporates the qualitative validity score approach instead of the commonly used uncertainty analysis. Al‐Washali et al. (2020) found that uncertainty analysis helps to improve the outputs of water loss assessment methods, although it did not demonstrate the accuracy or the validity of the methods. Even so, using the uncertainty analysis to improve the output of the tools is not questionable and strongly recommended (Alegre et al., 2016; AWWA, 2016; Lambert et al., 2014).

### 4.3 Water loss assessment approach

The approach used to establish the water balance in all the above nine tools, except CalcuLEAKator, is the top‐down water audit methodology, where AL are estimated and then RL are calculated. For CalcuLEAKator, the approach used is the MNF analysis (Equations 4, 5, 6) for one specific DMA and then water balance and NRW PIs are generated for this particular DMA. The tool enables data entry and water balances for 20 DMAs and based on these DMA mini‐balances, a global water balance, and NRW PIs for the whole network are compiled and created.

As can be noticed from the above description, the top‐down water audit is the main approach used to establish the water balance. The MNF analysis and BABE are usually used as complementary analyses for the top‐down water audit. However, using more than a method for establishing the water balance for the entire network is recommended (Al‐Washali et al., 2020), because it can improve the accuracy of the tool significantly and assist in establishing more reliable and system‐wide balances. For the DMA‐scale, MNF analysis remains a powerful methodology to establish the water balance in DMAs.

## 5 TOOLS FOR WATER LOSS REDUCTION PLANNING

Out of the 12 available tools, many tools touch on several aspects of planning for water loss reduction for the whole network: five tools provide guidance matrix for leakage reduction intervention, two tools accommodate economic analysis, and three tools indicate the opportunity of global pressure management.

### 5.1 Guidance matrices

The common guidance matrix is shown in Table 5. The matrix was developed for the World Bank Institute as a target matrix and a banding system for leakage performance categories. The limits of categories for low and mid‐income countries were set as twice the allowance of high‐income countries (Lambert, 2015a), to set feasible targets for water utilities in low and mid‐income countries. Having the volumetric leakage level or through the ILI, the leakage category of a certain utility is easily defined in Table 5. Based on the categories A1, A2, B, C, and D, different recommendations are provided (Liemberger and Partners, 2018):

**Table 5:** Leakage assessment matrix

			Leakage (L/connection/day) with *P*_avg_:				
		ILI	10 m	20 m	30 m	40 m	50 m
Standard	A1	<1.5		<25	<40	<50	<60
	A2	1.5–2		25–50	40–75	50–100	60–125
	B	2–4		50–100	75–150	100–200	125–250
	C	4–8		100–200	150–300	200–400	250–500
	D	>8		>200	>300	>400	>500
Low and middle income countries	A1	<2	< 25	<50	<75	<100	<125
	A2	2–4	25–50	50–100	75–150	100–200	125–250
	B	4–8	50–100	100–200	150–300	200–400	250–500
	C	8–16	100–200	200–400	300–600	400–800	500–1,000
	D	>16	>200	>400	>600	>800	>1,000

Source: EasyCalc v5.16.

Abbreviation: ILI, infrastructure leakage index.

A1: small potential for further NRW reductions; A2: further NRW reduction may be uneconomic unless there are water shortages or very high water tariffs; B: potential for marked improvements; establish a water balance, consider pressure management, ALC, better network maintenance, improve customer meter management, review meter reading, data handling and billing processed and identify improvement potentials; C: poor NRW record; tolerable only if water is plentiful and cheap; even then, analyze level and causes of NRW and intensify NRW reduction efforts; and D: Highly inefficient; a comprehensive NRW reduction program is imperative and high‐priority.

The matrix presented in Table 5 is provided in EasyCalc, BenchLoss, and CalcuLEAKator. EasyCalc provides this matrix but also another similar matrix for the total volume of NRW. CheckCalcs has a similar leakage matrix but with splitting the B, C, and D categories into two sub‐categories for each category, following practices in Australia and Malaysia, with exactly the same approach of A1 and A2 in Table 5. AWWA Water Audit has, in turn, preset targets for the ILI and coupled with technical and financial considerations. These matrices are useful and commonly applied, however, they are developed based on mere experience, not a scientific foundation nor published materials with deeply studied data.

### 5.2 Economic leakage detection

**Figure 2:**
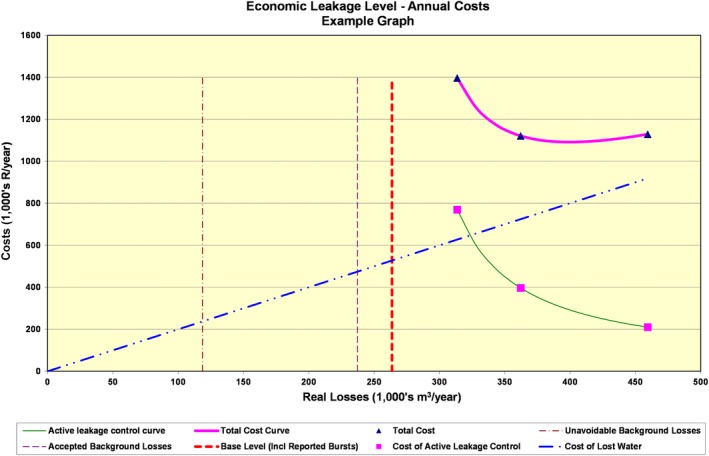
Economic level of leakage detection. Source: EconoLeak software v1.a

EconoLeak estimates the total volume of the leakage, background losses, and their costs. Afterward, it aggregates the leakage reduction cost in terms of sounding cost, leak correlation cost, MNF cost, repair cost, and finally administration and supervision cost. A simple cost–benefit analysis enables plotting of the curve of the short‐run ELL or the economic leakage detection. Figure 2 represents the main output of EconoLeak, where the *x*‐axis represents the leakage level and also the number of days required to survey the whole network using the leakage detection techniques. The ALC curve (green curve) shows that the detection survey becomes dramatically costly when the survey period of the whole system is less than 1 year and becomes more economic when it is more than a year (Mckenzie & Lambert, 2002). The lowest point in the total cost curve (pink curve) in Figure 2 corresponds to the economic survey period, which is almost annually in this example. The dotted vertical lines represent the base, economic and unavoidable levels of leakage of this example system. Any further leakage reduction after the economic (accepted) threshold in Figure 2 becomes basically uneconomic.

Obviously, this approach requires extensive cost data for all single elements of intervention. The tool in fact does not create the (long‐run) economic level of the leakage but only the economic intervention frequency of the leakage detection survey. It does not consider other interventions such as pressure management, assets management and response time minimization. The economic level of the leakage should be determined based on all possible intervention for a specific case and not only the leakage detection survey. Alternatively, the tool Component Analysis has a more matured feature of defining the economic intervention frequency. It defines how frequent should the leakage detection survey complete the entire network. This is estimated based on the variable cost of water, intervention cost, and also the rate of rise of unreported leakage, as shown in Equations 11, 12 (Lambert & Fantozzi, 2005).

(11) $$\mathrm{EIF}=\sqrt{0.789\times \frac{\mathrm{CI}}{\mathrm{CV}\times \mathrm{RR}}}$$

(12) EP=100×12/EIF

where EIF (months) is the economic intervention frequency through the leakage detection surveys, CI is intervention cost ($/Km), CV is variable cost ($/m^3^), RR is the rate of rise of unreported leakage (m^3^/Km mains/day/year), and EP is economic percentage of system to be surveyed annually. The limitation of Equation (11) is the variable RR, which is sensitive and difficult to estimate. However, guidance for estimating this factor is available in Fanner and Lambert (2009). Another concern of Equation (11) is its probable overestimation of the leakage detection potential, as discussed in AL‐Washali, Sharma, Kennedy, et al. (2019).

Interestingly, the tool Component Analysis does not only estimates the frequency of the leakage detection survey, but also gives an opportunity in doing cost–benefit analysis of pressure management and response time minimization for the whole network. Firstly, the tool enables setting the exponent of the pressure‐leakage relationship (N_1_; Equation 13) and then the tool estimates the potential volumetric and monetary savings of pressure reduction. It is worth mentioning that the leakage discharge from pressurized pipes varies with pressure during the day as highlighted by the concept fixed and variable area discharges (FAVAD; Lambert, 2001; May, 1994), which overcomes the limitations of Torricelli equation for plastic pipes. Equation (13) presents the leakage‐pressure relationship that is reconciled with intensive empirical research, using Japanese and UK data. The leakage exponent N_1_ in Equation (13) varies from 0.5 for rigid pipe materials to 1.5 for variable leakage area in flexible (plastic) pipe material, whose leaks' split varies with pressure.

(13) $$\frac{L_{1}}{L_{0}}={\left(\frac{P_{1}}{P_{0}}\right)}^{N_{1}}$$

where *L* is the leakage volume, *P* is the pressure, and N_1_ is the leakage‐pressure exponent. Similarly, the Component Analysis tool estimates the potential volumetric and monetary saving of minimizing the repair‐response time, based on the direct cut of the leaks' run‐time. Expectedly, the tool does not recognize the economic aspect of assets management, perhaps because estimating the benefits of the assets management is complex and it is typically not cost‐effective. This tool is probably the most economically comprehensive tool for leakage management that is freely available. Example of the output of this tool is presented in Al‐Washali et al. (2020) and AL‐Washali, Sharma, Kennedy, et al. (2019).

### 5.3 Global pressure management opportunity

There are many network simulation and hydraulic modeling tools that consider pressure management and pressure control for water networks. This is basically based on the concept that optimizing the pressure in the network triggers significant leakage reduction. Incorporating pressure management with DMA demarcation is commonly utilized by such tools. An example of these tools is the freely available tool, EPANET and its possible add‐ins modules. Several (commercial) tools are also available, including: WaterGYMS, InfoWater, WDNetXL, H_2_O MAPWater, KYPIPE, and other tools. However, there are three freely available tools that snapshot the pressure management opportunity for the whole (global) network and estimate the feasibility of pressure management without the need of hydraulic simulation of the network pipes and appurtenances, which is the focus of this review. These tools are: CheckCalcs, Component Analysis, and EasyCalc. The principle is similar and intuitive. Based on defining the exponent of the leakage‐pressure relationship (N_1_) in Equation (13), the reduction on the leakage volume as a consequence of reduced pressure can be estimated, and then its monetary value can be easily derived. This is what EasyCalc exactly does. However, CheckCalcs further analyses the impact of pressure reduction on the reduction of bursts frequencies as well as the customers' consumption (N_2_ and N_3_, respectively). The Component Analysis, however, enables estimating the benefit of pressure management and comparing it to its cost as well as the costs and benefits of other leakage interventions.

## 6 TOOLS FOR WATER LOSS MANAGEMENT INTERVENTION

The previous two sections tackle the assessment of water losses and planning for its management. However, when it comes to working on the ground during the intervention phase, only the tools that are focused on DMA‐scale are hands‐on. These tools are SanFlow and PresMac.

### 6.1 Active leakage detection

**Figure 3:**
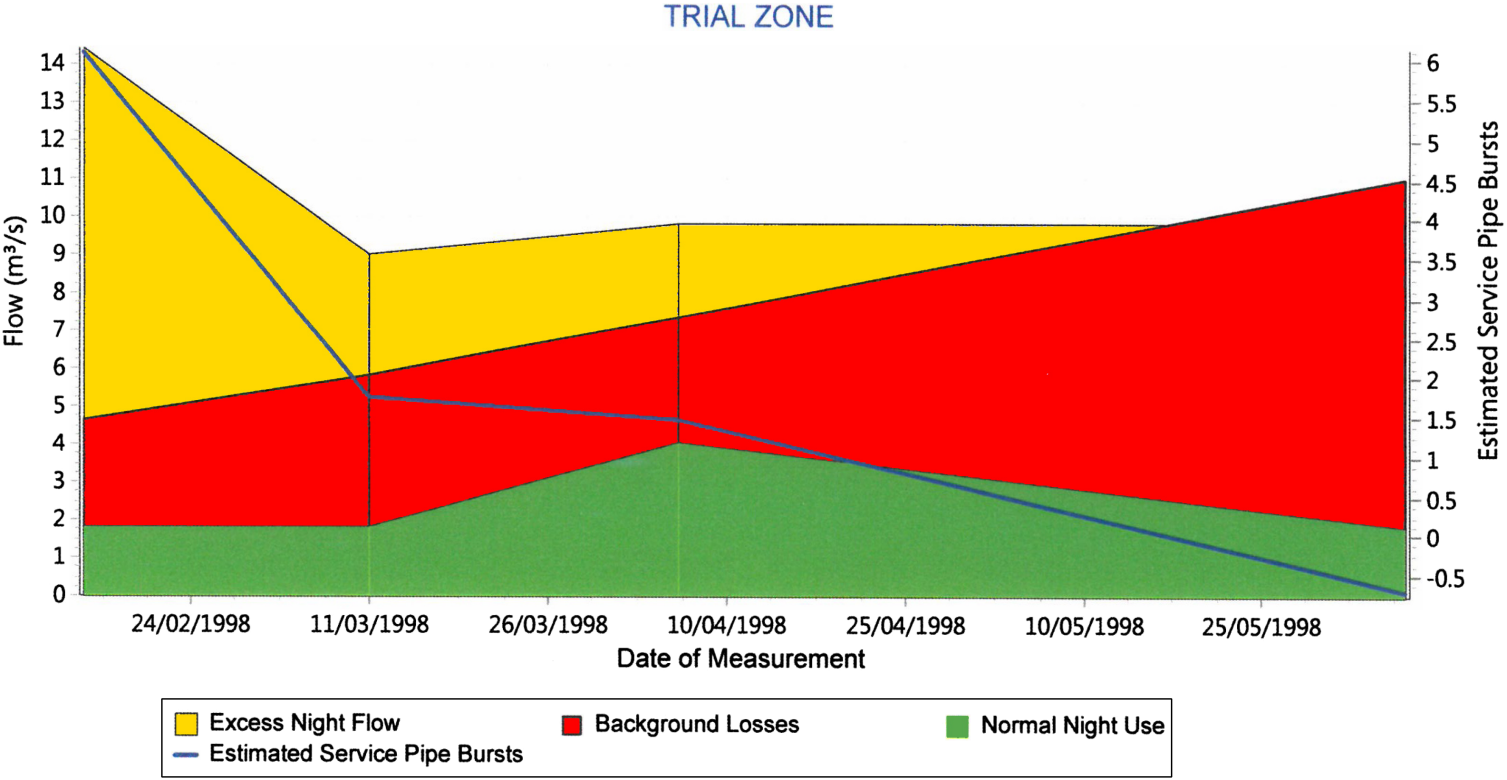
Leakage, background losses, and night use in an example DMA. Source: SanFlow v4.6. DMA, district metered area

Similar to CalcuLEAKator which is discussed in Section 4, SanFlow is a powerful tool that also analyses MNF in a DMA within the network using Equations 4, 5, 6. It is a suitable and operational tool to intervene and reduce the leaks in a DMA. It estimates the legitimate night use, the leakage and the unavoidable background losses using the BABE approach. After deducing the unavoidable background losses, the tool transforms the leakage volume for each DMA into an estimated equivalent number of bursts in the DMA. Notably, the tool does not assume the presence of bursts within each DMA, but use this as an index to rank the DMAs based on their leakage level. Comparing this equivalent number of bursts for all the DMAs assists in prioritizing the DMAs for leakage minimization interventions. SanFlow estimates the hourly leakage only during the MNF time and does not provide estimates on a daily leakage level, let alone annually. Figure 3 gives an insight into the output of SanFlow. Figure 3 implies that there was significant leakage in the DMA at the beginning (the yellow area) and after some time, when the leaks were fixed, the pressure in the DMA enhanced, causing more unavoidable background leakage in the DMA that cannot be sensed by the available leakage detection technology. So the blue line, which shows the estimated equivalent service pipe bursts, represents the priority level of intervention in this DMA, and can be compared with other DMAs in the network which is a main function in this tool.

### 6.2 Zonal pressure management

**Figure 4:**
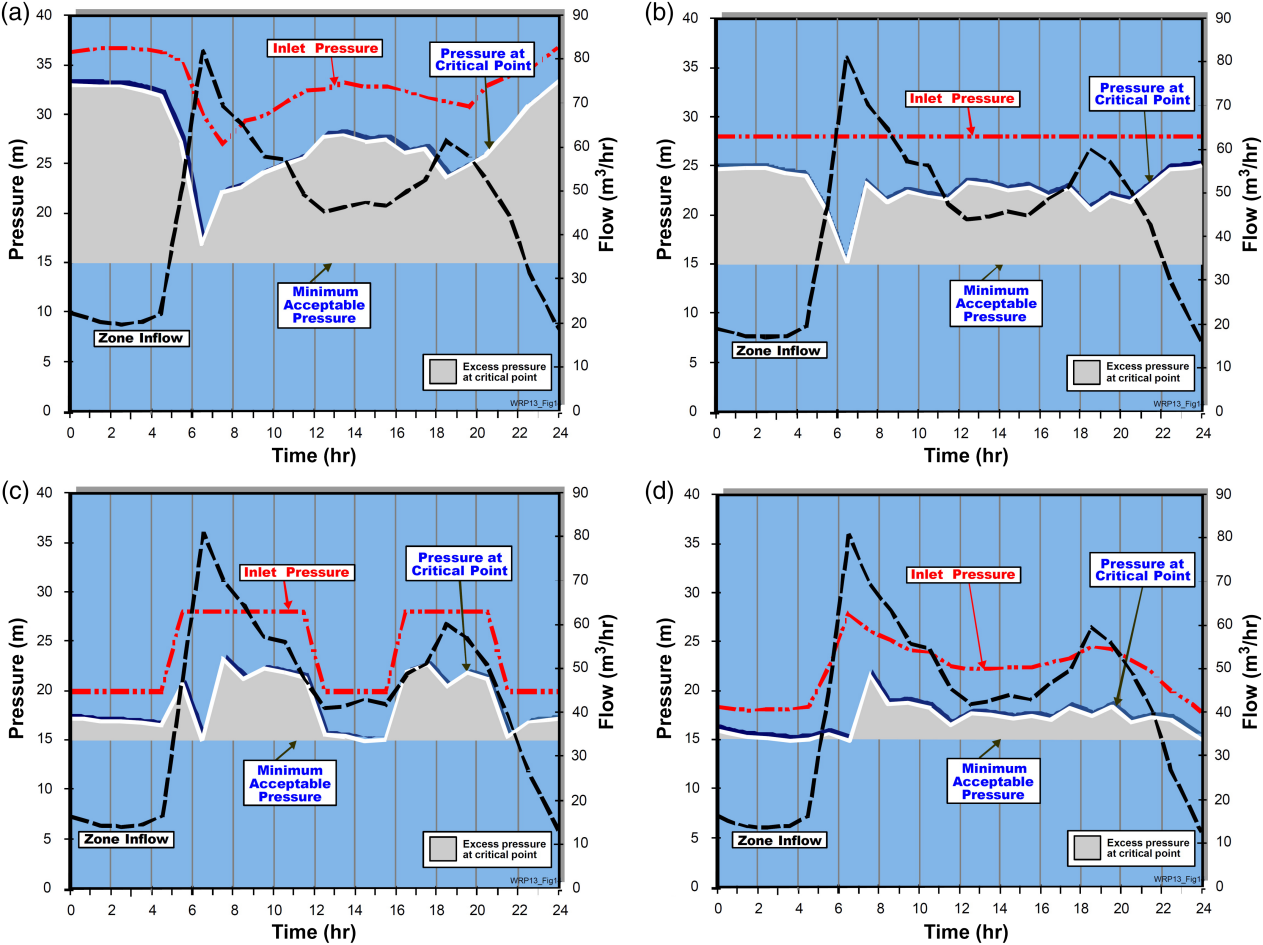
Pressures at a critical point in a DMA: (a) no PRV, (b) a fixed‐outlet PRV, (c) a time‐modulated PRV, and (d) a flow‐modulated PRV. Source: manual of PresMac v4.4. DMA, district metered area; PRV, pressure reducing valve

The potential for pressure management is consistently promising. Reducing the pressure at a critical point in a DMA by a certain level triggers a more pressure reduction at the inlet of the DMA (Mckenzie & Langenhoven, 2001). PresMac, therefore, assesses the monetary savings of pressure reduction due to installing fixed‐outlet and time‐modulated PRVs in a specific DMA. The tool does not rely on hydraulic representation of the DMA's network, rather, it compromises MNF analysis, BABE analysis, N_1_ exponent estimation, and further analyzing pressure measurements, friction factors (*K*) and head losses (*H*_L_) in three key points in the DMA: the inlet point, the average (elevation) zone point, and the critical point(s) in the DMA whose pressure is the lowest in the DMA during the course of the day. The fixed‐outlet PRV dictates a fixed pressure during all the hours of the day (Figure 4b). The time‐modulated controller reduces the outlet pressure at certain times of the day when the demand is basically reduced (Figure 4c). In a critical point modulation, the pressure is sensed at the critical point and communicated to the PRV at the inlet which, in turn, adjusts the inlet pressure to maintain the minimum pressure at the critical point during the course of the day. This process is effective, but further investment is required. In PresMac, the flow‐modulated controller (Figure 4d) dictates the inlet pressure in accordance with the instantaneous demand and the excessive pressure at the critical point in the DMA. While firefighting flows cannot be met using the time‐modulated controller, it can be satisfied using the flow‐modulated controller. The use of PRVs optimizes the pressure in the DMA and achieve the minimum leakage. PresMac, however, analyses only the benefits of fixed‐outlet and time‐modulated PRVs. If the less expensive time‐modulated controller is economically justified, the flow‐modulated controller can provide greater benefits. A limitation of pressure reduction is the potentially small reduction in customer demand. In this regard, PresMac assumes that leaks are pressure‐dependent and consumption is pressure‐independent, although some consumption is in fact pressure‐dependent, such as washing hands, brushing teeth and garden irrigation.

The approach used in PresMac is iterative. Firstly, using MNF measurements and BABE analysis, the tool estimates the background leaks and solves Equation (13) to estimates the value of N_1_ as shown in Equation (14). Secondly, the tool splits the inflow into the DMA into pressure‐dependent and pressure‐independent flows. Then, the tool estimates the friction factor (*K*) using Equation (15) for each hour of the day at two points: the average zone point and the critical point in the DMA.

(14) $$N_{1}=\frac{\log\left(\frac{L_{0}}{L_{1}}\right)}{\log\left(\frac{P_{0}}{P_{1}}\right)}$$

(15) $$K=\frac{H_{L}}{Q^{2}}$$

where *K* is head loss coefficient (m^−5^ hr^2^), *H*_L_ is head loss in m, and *Q* is flow in (m^3^/hr). The tool then selects a fixed‐outlet pressure and recalculates pressure in the average zone point and the critical point using *K* factors and the corresponding zone inflow. This process is iterative until the minimum acceptable pressure at the critical point is achieved. This process is carried out for the fixed‐outlet PRV which saves significant leakage volume. Nonetheless, the achieved minimum acceptable pressure at the critical point is only for the peak hour and is still higher than the required pressure during the remaining hours of the day, when the demand is lower. Therefore, the time‐modulated controller can further reduce the leakage by changing the times and switching from high to low‐pressure periods during the day, and the benefits of this option are particularly analyzed in PresMac. The benefits of adopting flow‐modulated PRV in the DMA are unfortunately not provided in PresMac. Finally, it is worth mentioning that recent researches on N_1_ exponent (Lambert, Fantozzi, & Shepherd, 2017; Van Zyl & Cassa, 2014) revealed that N_1_ itself is affected by the changing pressure during the day and this particular issue is still not considered in PresMac.

## 7 SUMMARY OF THE TOOLS' MODULES AND GAPS

Table 3 summarizes the modules and gaps for each individual software tool. The functions of these tools fall into the above three categories: (a) water balance establishment; (b) water loss reduction planning; and (c) water loss management intervention. Although asset management is a main pillar of RL reduction, it is still not recognized in the NRW reduction tools. Besides, while AL is a major concern in low‐ and mid‐income countries, it is not sufficiently recognized in the input data for most of the tools. Providing more detailed input for AL results in more accurate results. Table 4 shows the level of details of the tools in terms of AL and other key parameters that affect the accuracy of the water balance. Table 4 shows that only one tool, the WB‐EasyCalc, has a sufficiently detailed module for AL. Regarding AL reduction, Table 3 shows that AL reduction planning has garnered relatively little attention among software developers, which is a major limitation in the industry in general. A good start in this regard would consist of developing a tool or a module to optimize policies for customer meter replacement in the distribution network. It is worth to highlight that the reviewed tools are mainly developed by practitioners in the field, aiming to be applicable in real‐world and for different network sizes. The reviewed tools use hydraulic analysis and hydraulic modeling to a certain extent, however, they do not incorporate hydraulic representation of the network's pipes and appurtenances neither for the whole network nor for a DMA in the network.

## 8 GUIDANCE FOR THE USE OF NRW SOFTWARE TOOLS

**Figure 5:**
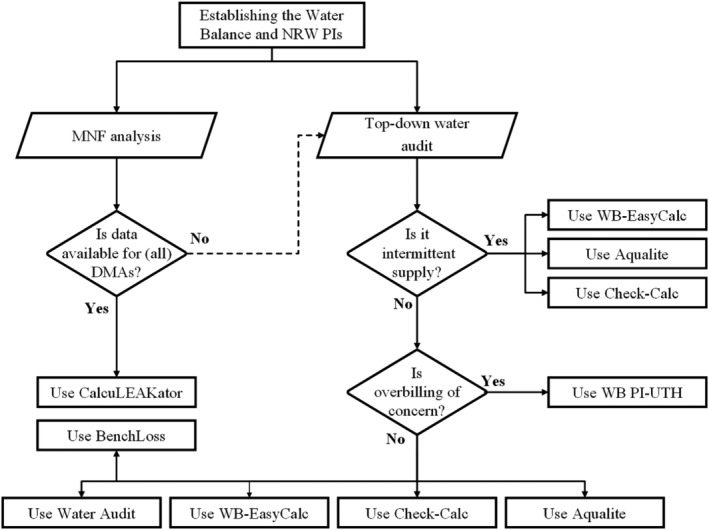
Flowchart on the use of NRW assessment software tools. NRW, nonrevenue water

Figure 5 presents a fit‐for‐purpose guide for selecting an NRW software tool to establish the water balance and generate NRW PIs. When using the top‐down water balance, if the water supply is intermittent, then three tools can be used: WB‐EasyCalc, Check‐Calc, and Aqualite. If the water supply is continuous and overbilling of consumption is of concern, then WB‐PI Calc‐UTH can be used. Otherwise, Water Audit, WB‐EasyCalc, Check‐Calc, Aqualite, and BenchLoss can be used. When the water balance needs to be established using MNF, then CalcuLEAKator can be used.

**Figure 6:**
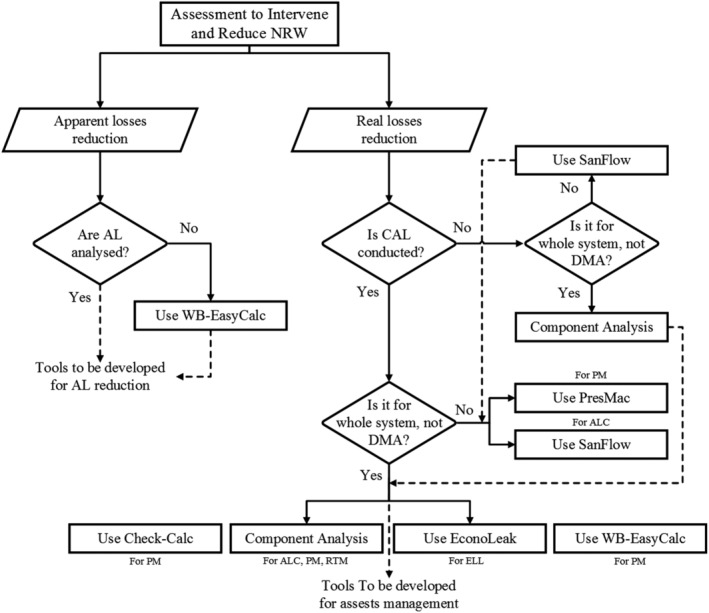
Flowchart on the use of NRW intervention software tools. NRW, nonrevenue water

Similarly, Figure 6 presents a fit‐for‐purpose guide for selecting an NRW software tool when assessing water loss to plan for reduction measures. Planning for RL reduction requires conducting a BABE analysis (i.e. CAL). Planning for RL reduction can be accomplished using two scales: the whole network or within a DMA. For the whole network, a BABE analysis can be conducted using the tool Component Analysis. To analyze the potential for leakage reduction when carrying out pressure management interventions, the tools Component Analysis, Check‐Calc, and WB‐EasyCalc can be used. To analyze the ELL, EconoLeak can be used. To analyze the potential for leakage reduction when minimizing the response times for reported bursts or when carrying out active leakage detection, the Component Analysis tool can be used. To plan for RL reduction on a DMA‐scale, Sanflow is first used to conduct a BABE analysis. Afterward, Sanflow is again used to prioritize between DMAs for active leakage detection and then PresMac can be used for pressure management analysis. Unfortunately, planning for AL reduction is still missing and tools need to be developed for this purpose.

## 9 CONCLUSIONS AND FUTURE PROSPECTS

The availability of several NRW software tools and many iterations of each individual tool indicate the promising future of NRW software development. This article reviewed 12 freely available NRW software tools for assessing NRW in distribution networks. The tools use hydraulic analysis in their modules, but they do not hydraulically model a representation of the network's pipes and appurtenances. Most (nine) of the tools have been developed to establish the standard annual water balance and NRW recommended PIs for the whole network. Some (three) tools, however, have been developed to intervene and reduce leakage within a DMA.

The main aspects of the software tools that are currently improving or need to be improved further in the software tools are: uncertainty analysis, consideration of supply intermittency, and loss reduction analyses. Although the importance of uncertainty analysis is widely recognized in the industry, only five tools have a module for uncertainty analysis. Intermittency of the water supply is a subject of increasing interest, yet it is considered in only four tools. Whenever intermittency is of concern, normalizing the PIs to a continuous supply is necessary, and this has been included in only four tools (which consider only real losses). Normalizing the NRW and apparent losses remain unaddressed. To plan for leakage reduction, the tool Component Analysis Model is relatively comprehensive as it analyses the potential benefits of pressure management, active leakage detection, and minimizing response and repair time of bursts. However, apparent losses have still not been adequately recognized in the industry.

Although a comprehensive NRW management tool for monitoring, planning, and intervention is currently not available, such a tool can be developed in one complete package or in a kit of several tools. The modules presented in Table 3 should be included in such a tool. However, certain critical aspects should be emphasized. Firstly, recognizing the intermittency of the supply will widen the use of NRW software tools. For this normalizing all the volumes and PIs of the NRW, apparent losses, and real losses is essential. Currently, normalization is only considered for real losses and not for apparent losses and NRW. Secondly, distinguishing the scales of the tool is crucial. Planning for loss reduction should first be performed for the whole network; then planning for specific interventions should be done on a DMA‐scale. In both scales, a comprehensive tool that considers different reduction measures for both real and apparent losses should be useful and effective. Thirdly, greater focus needs to be placed on the apparent loss estimation and minimization; the reduction of apparent losses is cost‐effective and it is relevant to, and considered a priority for, many water utilities in low‐ and mid‐income countries. Fourthly, a comprehensive tool for NRW would definitely benefit from including capabilities for uncertainty analysis, guidance matrix and assessing NRW components using different methods. Finally, NRW software tools should include the capability to determine the economic level of water loss based on estimating and combining the economic levels of leakage and apparent losses.

## CONFLICT OF INTEREST

The authors have declared no conflicts of interest for this article.

## AUTHOR CONTRIBUTIONS

**Taha Al‐Washali:** Conceptualization; data curation; formal analysis; investigation; methodology; software; supervision; validation; visualization; writing‐original draft; and writing‐review and editing. **Mohammed Elkhider:** Data curation; formal analysis; investigation; methodology; software; visualization; and writing‐original draft. **Saroj Sharma:** Conceptualization; formal analysis; investigation; methodology; project administration; supervision; validation; and writing‐review and editing. **Maria Kennedy:** Conceptualization; funding acquisition; project administration; resources; supervision; validation; and writing‐review and editing.

## RESEARCH RESOURCES

The reviewed tools for nonrevenue water management.

## RELATED WIREs ARTICLES

https://doi.org/https:/doi.org/10.1002/047147844X.mw1817

https://doi.org/https:/doi.org/10.1002/stc.187

https://doi.org/https:/doi.org/10.1111/j.1747-6593.1998.tb00173.x

https://doi.org/https:/doi.org/10.1111/j.1747-6593.2000.tb00248.x

## Supporting information


**Appendix** S1. Supporting Information
