# Supplementary material for: A review of nonrevenue water assessment software tools
Source: WIREs Water. 2020 Feb 18;7(2):e1413. doi: 10.1002/wat2.1413 (PMC7074021; doi:10.1002/wat2.1413)
Supplement: Supplementary file 1 — Appendix S1. Supporting Information [file WAT2-7-e1413-s001.docx]

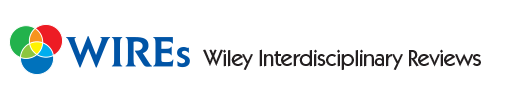


**A Review of Non-Revenue Water Assessment Software Tools**

**Article Type:**

**Authors:**

| **Taha M. AL-Washali***  Department of Civil Engineering and Geosciences, Delft University of Technology, Stevinweg 1, 2628 CN Delft, The Netherlands; Environmental Engineering and Water Technology Department, IHE Delft Institute for Water Education, Westvest 7, 2611 AX Delft, The Netherlands; Water and Environment Center, Sana’a University, Yemen; t.m.y.al-washali@tudelft.nl; t.alwashali@un-ihe.org |
| --- |
| **Mohammed E. Elkhider**  Environmental Engineering and Water Technology Department, IHE Delft Institute for Water Education, Westvest 7, 2611 AX Delft, The Netherlands; eng.mohamed_elkhider@hotmail.com |
| **Saroj K. Sharma**  Environmental Engineering and Water Technology Department, IHE Delft Institute for Water Education, Westvest 7, 2611 AX Delft, The Netherlands; s.sharma@un-ihe.org |
| **Maria D. Kennedy**  Department of Civil Engineering and Geosciences, Delft University of Technology, Stevinweg 1, 2628 CN Delft, The Netherlands; Environmental Engineering and Water Technology Department, IHE Delft Institute for Water Education, Westvest 7, 2611 AX Delft, The Netherlands; m.d.kennedy@tudelft.nl; m.kennedy@un-ihe.org |

**Supplementary information about the NRW assessment tools**

1. **Aqualite**

Aqualite is a Windows-based tool developed by Mckenzie (2007) and the Water Research Commission (WRC) in South Africa to establish the top-down water balance and NRW PIs. It is an updated version of the tool BenchLeak. Aqualite can use different units and the intermittency of the supply and uncertainty analysis are included in the tool. However Aqualite normalises only the PIs of the RL, assuming that the supply is continuous. Volumes and PIs of NRW and AL remain unnormalised and are therefore affected by the level of intermittency and the changes in the duration of the supply.

1. **AWWA Water Audit**

Water Audit is an Excel-based tool developed by the American Water Works Association (AWWA) (WLCC 2014) to establish the water balance and NRW PIs. A unique feature of this tool is the use of validity scores and guidance instead of uncertainty analysis. The validity guidance triggers changes in data acquisition rules when a low validity score is recorded. However, it is questionable why the tool uses the qualitative validity score approach instead of the commonly used uncertainty analysis. Al-Washali et al. (2020) found that uncertainty analysis helps to improve the outputs of water loss assessments, although it did not demonstrate the accuracy or the validity of the method. Another feature of this tool is its use of a guidance matrix for the input data to plan water loss control. However, this tool does not consider the intermittency of the supply and therefore all NRW PIs are not normalised. Moreover, it provides limited details for the AL, i.e. it uses one figure for each component of AL, without providing for the possibility of more details.

1. **BenchLeak**

This is an Excel-based tool developed by the WRC to establish a water balance and compute NRW PIs (Mckenzie et al. 2002). Its functionalities are very basic and as an initial tool for analysis, has been substituted by AquaLite. However, BenchLeak remains freely available for generating the NRW PIs for water utilities in South Africa. A good feature of this tool is the associated user manual which clarifies the concepts behind the calculations made in the tool. However, BenchLeak is, in general, outdated.

1. **BenchLoss**

BenchLoss NZ is an Excel-based tool developed by Global Water Resources (GWR) for water utilities in New Zealand (GWR-Ltd 2008). The tool assists in establishing the water balance and NRW PIs and benchmarks the performance relative to other utilities in New Zealand. BenchLoss intensively explains the input and the output of the tool and identifies appropriate action priorities based on a guidance matrix. The tool estimates the confidence limits of the inputs and the uncertainties of the outputs. It also assesses the details of AL components. However, BenchLoss does not consider the intermittency, normalisation, and other features of leakage reduction.

1. **CalcuLEAKator**

This is an Excel-based tool designed by an independent consultant (Koldžo and Vucˇijak 2013) to analyse and compile MNF analyses. The tool can generate the water balance and NRW PIs based on both top-down and bottom up approaches, which is a unique feature of the tool. However, establishing the water balance for the whole network based on MNF data or measurements of some DMAs is always questionable, because MNF analysis in one or several DMAs during a specific period will always be different from other parts of the network and at other times of the year.

1. **Component Analysis Model**

Component Analysis is a more comprehensive Excel-based model developed by the AWWA Water Research Foundation (WRF) (Sturm et al. 2014). It establishes the water balance and NRW PIs and analyses the components of the RL using the CAL method for the whole network. It then analyses the potential and the economic feasibility of reducing the leakage through pressure management (PM), active leakage detection and control (ALC), and repair response time minimisation (RTM). Component Analysis was developed for water utilities in North America and therefore, has a benchmarking feature to other utilities in North America. However, this tool does not consider intermittency, uncertainties, and normalisation.

1. **EconoLeak**

This is an Excel-based tool developed by the WRC for determining the economic level of leakage (ELL) (Mckenzie and Lambert 2002) by plotting the curve of the ELL based on cost and benefit estimation of ALC in the network. The tool has a user guide explaining the principles behind the calculations of the model. However, the tool was developed for water utilities in South Africa and therefore uses local currency and cost estimates.

1. **CheckCalcs**

CheckCalcs is an Excel-based tool developed by ILMSS Ltd for establishing the water balance and NRW PIs (Lambert 2015a). It includes detailed instructions, and it benchmarks the system being analysed to 12 other European systems; and it provides a guidance matrix to identify appropriate action priorities. In addition, this tool gives insights on the probable changes in leaks, bursts, and consumption (N_1_, N_2_, and N_3_, respectively) when the pressure of the system is changed. However, this model accepts limited details and inputs for determining the AL.

1. **PresMac**

PresMac is a Windows-based tool developed by the WRC as an operational tool for pressure management in a DMA (Mckenzie and Langenhoven 2001). PresMac includes a guidance manual explaining the concepts of the tool. The tool features fixed outlet as well as time-modulated analysis for pressure reducer valves (PRVs); it is the only free pressure management tool that works on a DMA scale. PresMac considers both the pressure-dependent and -independent flows for leaks and for legitimate night uses. A unique feature of this tool is its ability to calculate the value of the pressure-leakage relationship exponent (N_1_) based on night-time inflow and pressures within a DMA. While estimating the relationship between the leakage exponent N_1_ and the fluctuating pressure in a DMA during the day is a matter of increasing concern (Lambert et al. 2017; Van Zyl and Cassa 2014), this is not considered in the model.

1. **SanFlow**

SanFlow is a Windows-based tool developed by the WRC to analyse night flows using the MNF analysis approach in a DMA (Mckenzie 1999). It also breaks down the RL in the DMA into its component parts using the CAL. However, SanFlow does not calculate the uncertainties associated with the outputs. It also estimates the hourly leakage only during the MNF time and does not provide estimates on a daily leakage level. Instead, the tool transforms the leakage volume during the MNF hours into equivalent estimated bursts. Comparing this equivalent number of bursts for all the DMAs assists in prioritising the DMAs for leakage minimisation interventions.

1. **WB-EasyCalc**

WB-EasyCalc is an Excel-based tool developed by Liemberger and Partners (2018) to establish water balance and NRW PIs. This tool has many advantages. It is tidy and user-friendly; it provides details for all the components of the water balance and considers the uncertainty of the outputs. It also recognises intermittency of supply and therefore has normalised PIs for the RL. However, the volumes of the NRW and AL and their PIs are unnormalised in this tool. The tool features a ‘’what if’’ scenario analysis when changing the pressure or the duration of the supply in the network. It also enables historic water balance data comparison. However, the tool would definitely benefit from the inclusion of the component analysis of the RL and consideration of the overbilling practices in the system.

1. **WB-PI Calc-UTH**

This is an Excel-based tool developed by Tsitsifli and Kanakoudis (2010) to establish water balance and NRW PIs. This is the only tool that considers overbilling practices in the water balance. However, the tool is in Greek; and while it considers 170 PIs that may be suitable for the local context, these are not necessarily suitable for other utilities.
